# Laughter

**Published:** 1891-05

**Authors:** 


### LAUGHTER.

Tears and laughter are part of the universal language of human kind—the language of *looks*. Since Babel, men, dispersed over the face of the world, do not understand one another's speech. But this one inarticulate language remains intelligible to all mankind; it requires no interpreter, it is legible to those to whom even "the three R's" are still a mystery; infants newly-born seem to bring some understading of it with them into the world; it may be read by a black man in the face of a white; it would have been apprehended in Robinson Crusoe, by his man Friday on the desert island; it is even in some degree legible by a marvelous instinct by the lower animals in the face of man. For a strong man can flash a spirit-quelling command out of the bodily windows of his soul, his eyes, into the half-waking "spirit of a beast." A Borrow can thereby assert his authority "by right divine" over an enraged dog. It is a touching proof of that deep underlying unity, which, amidst all their infinite differences, binds together in the deeper regions of their being all the far separated races and families of Adam's children, that all men do in this way, understand one another's looks. If I land on an unknown island, whose inhabitants speak a language in the ordinary sense of that word quite unintelligible to me, and yet see there a human *face*, whatever be its color or shape, lighted with a smile, or trembling into tears, my heart, if it is not dead within me, will answer to what I call its *expression*, though I cannot in the least tell how I gather any such knowledge from that sight. We may plead, therefore, for the deep interest of these two phenomena, tears and laughter, on this ground among others, that they are part of the universal and distinctive characteristics of our brother men, of every race and clime. Laughter is visible principally in that mystic borderland between matter and spirit, "the human face divine." How it does so, who shall say? For what is a face? It is a region but a few square inches in extent; and yet this is all the instrument, or much the principal one, with which in the mystic process of Nature, all the varieties of thought, feeling, emotion, of which we become aware in looking at another human being, are in some way or other effectively conveyed to us. Its component parts have, it is true, a marvellous power of ceaseless, most subtle *movement*—a most important attribute. "That," says Lord Bacon (essay on Beauty) "is the best part of beauty which a picture cannot express; no, nor the first sight of the life, decent" (*i. e.*, becoming) "and gracious motion." Still, it is thought this apparently simple instrument, a face, that in some way or other, thought, feeling, emotion, are expressed to a degree really marvellous. And who knows not the actual light (is it physical, or is it spiritual, in its essence? we cannot say) that may flash into our souls from the lines of flowing round the mouth, the dancing gleams in the lengthening eyes; the innumerable twinklings and beamings of the countenance, when it is really *laughing*.

Still, it is not only in the face, or even in the domain of sight, that the spiritual condition which causes laughter is perceptible in others. The blind, who never looked upon a face, yet know of laughter in others through that other bodily doorway into the presence chamber of the soul, the ear; and the feelings called up in the soul of the blind by peals of laughter (*peals* as of some gladsome and brilliant bells in the spirit-world), must be much the same as those stirred in those deprived of hearing by the sight of a laughing face, and of the shaking sides—the arms, it may be, flung into the air, the head thrown back, or the hands enthusiastically rubbed together—of one who is undergoing that strange seizure rightly called a fit—for a true fit or physical seizure it is—of laughter. Whatever it may be in its inmost nature and central spring in the soul, its effects are general over the whole body. Probably there is not the remotest corner, or little inlet of the minute blood-vessels (life-vessels) of the body, that does not feel some wavelet from great convulsion shaking the central man. The blood moves more lively—probably its chemical, electric, or vital condition is distinctly modified—it conveys a different impression to all the organs of the body as it visits them on that particular mystic journey when the man is laughing, from what it does at other times. And so, we doubt not, a good laugh may lengthen a man's life, convey a distinct stimulus to the vital forces. And the time may come when physicians, attending more closely than at present unfortunately they are apt to do, to the innumerable subtle influences which the soul exerts upon its tenement of clay, shall prescribe to a torpid patient "so many peals of laughter, to be undergone at such and such a time," just as they now do that far more objectionable prescription, a pill, or an electric or galvanic shock; and shall study the best and most effective method of producing the required effect in each patient.—*The Family Doctor.*

